# Bonding Performance for Repairs Using Bulk Fill and Conventional Methacrylate Composites

**DOI:** 10.1155/2021/2935507

**Published:** 2021-12-16

**Authors:** Janaina Galvão Benzi, César Rogério Pucci, Maiara Rodrigues Freitas, Priscila Christiane Suzy Liporoni, Rayssa Ferreira Zanatta

**Affiliations:** ^1^Department of Restorative Dentistry, School of Dentistry, Taubaté University, UNITAU, Department of Dentistry, Taubaté, Brazil; ^2^São Paulo State University-UNESP, Institute of Science and Technology, Department of Restorative Dentistry, São José dos Campos, São Paulo, Brazil; ^3^Department of Dentistry, School of Health Sciences, University of Brasília, Brasilia, Brazil

## Abstract

This study compared the bond strength of a composite repair made with a bulk fill composite and a conventional one using different surface treatments. Specimens were prepared as truncated cones (bases: 4 mm × 2 mm, height: 4 mm) using a bulk fill (OBFa: Filtek One) or a conventional resin (FTKa: Filtek Z250) (*n* = 66). They were artificially aged (10,000 cycles, 5°C–55°C, 30 sec) and subdivided according to surface treatments: NT—no treatment (control), Abr—abrasion with a diamond tip, and sand—sandblasting with aluminum oxide (50 *μ*m). Treatments were performed over the smaller diameter surface, followed by adhesive (Scothbond Universal) application. A new specimen with similar dimensions was constructed over it using either the OBF or the FTK, totaling 12 groups (*n* = 11). Bond strength was assessed by tensile test. The data were submitted to two-way ANOVA separately for OBFa and FTKa, followed by Tukey's test (*p* < 0.05). For the aged OBFa groups, there was significant differences for composite type and surface treatment, with higher values of bond strength when repaired with the same material (OBFa/OBF > OBFa/FTK), and sandblasting and bur abrasion presented higher values compared to the control group (NT). For the aged FTKa groups, there were no differences for the composite or surface treatment. Therefore, the bulk fill resin composite tested present better repair performance when the same composite was used, while the conventional resin composite was less influenced by the material and the surface treatment performed.

## 1. Introduction

In the 1960s, the PMMA (polymethyl methacrylate) was the main monomer employed for dental resin-based composite, but after that, bis-GMA (bisphenol-A glycidyl methacrylate) resin was introduced [1, 2], and since then, the composition of the composite resins has improved in terms of biomechanical properties and behavior, as well as optical, physical, and chemical characteristics, in order to mimic the dental structure [2, 3]. Nowadays, the adhesive strategies allow the work in a minimally invasive philosophy, and clinical studies show that direct restorative treatment using conventional composite resins has success rates greater than 80% in 10 years of follow-up [4–6] making them safe to use in long-term periods [5, 6].

Attempting to overcome problems associated with the conventional methacrylate composites, such as polymerization shrinkage, the bulk fill resin composites were launched on the market, with changes in the monomer's composition and in the translucency [7]. The new photoinitiators allows the use of increments up to 5 mm in thickness [8–10], and novel monomers guarantee reduced polymerization shrinkage compared to conventional methacrylate composite resins [10–12]. In addition, the insertion of larger increments in the cavity reduces the working time, the risk of contamination of the material, and errors during the procedure, making it an attractive option for clinical use [8–10]. Previous data show that bulk fill resins have satisfactory longevity in short and medium term [13–15], with reported failure rates around 5.6%, associated with secondary caries, fractures, marginal alterations, and postoperative sensitivity [15].

Within the minimally invasive philosophy treatments, the repair of early failures of direct restorative systems enables the restoration to be recovered, without its complete exchange, since the total removal of the restoration is accompanied by the removal of healthy dental tissue and increased cavity preparation [16]. Direct repair is considered an alternative, conservative, fast, low-cost treatment, which makes it possible to recover the restoration with noncritical partial defects, such as cases of marginal defects, anatomically shaped defects, roughness and unfavorable pigmentations, fracture, and wear of the material [8, 17]. However, bulk fill composite formulation includes different monomers and additives, some of which are unknown, that could diminish their potential repair [18].

Over time, the composite restoration becomes saturated with saliva, which removes the free radicals available in the surface and make it impossible to chemically react with the new composite in a repair [19]. It makes necessary to do a surface treatment in the former restoration to the new one, mainly due to the lower number of available C = C to react with the new material [18].

The surface treatment that is done before the repair restoration has two purposes: the first one is to remove the altered surface layer by exposure to saliva and by the aging of the restoration, in addition to increasing the surface energy of the outer layer of the resin, and the second is to increase the surface area and creating irregularities [20]. Brosh et al. [21] described that the union between the aged resin and the new one used in the repair can occur in three ways: (1) through the chemical union with the organic matrix; (2) through chemical bonding with the exposed charge particles; and (3) through micromechanical retentions of the treated surface.

Thus, the aim of the present study was to compare the bond strength of repairs using conventional and bulk fill one resin composites, using different surface treatments. The tested null hypotheses were H_1_: there was no difference in adhesive strength between the resins tested and the one used in the repair; H_2_: there was no difference between the surface treatments tested.

## 2. Materials and Methods

### 2.1. Specimen Preparation

**Figure 1:**
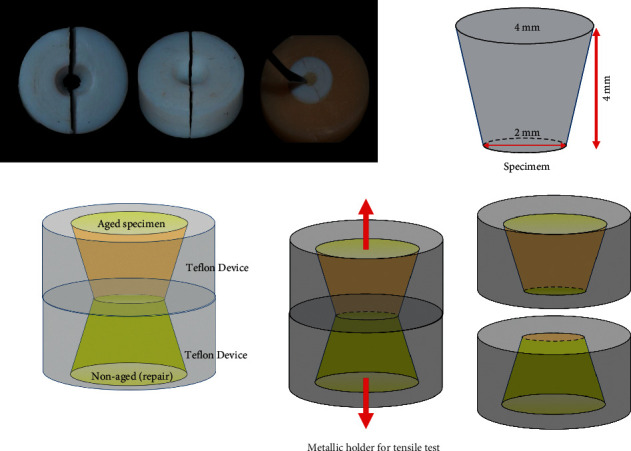
Schematic drawing of the specimen preparation.

Sixty-six artificially aged specimens were made in the shape of a truncated cone with help of a two-piece Teflon device, as previously described [22, 23] using a bulk fill resin composite (OBFa-Filtek One, shade A2-3M ESPE, St. Paul, USA), and other sixty-six were made with a conventional methacrylate resin composite (FTKa-Z250, shade A2-3M ESPE, St. Paul, USA). The specimens presented the larger base measuring 4 mm in diameter and the smaller one with 2 mm and 4 mm in height (Figure 1). Sample size calculation was performed using the Statistica software for Windows (v. 9.0, Statsoft, Tulsa, USA) considering the results of our pilot study, and was set on *n* = 11. Figure 1 shows the Teflon device and a schematic drawing of the samples.

The bulk fill resin was inserted in the Teflon device in a single increment of 4 mm, whereas for the conventional composite, it was inserted in 2 increments of 2 mm each. Both composites were cured from the larger diameter face with LED light (irradiance of 1400 mW/cm^2^-VALO, Ultradent, Vivadent, Schann, Liechtenstein), for 20 seconds.  Table 1 shows the composition of the materials used in the study.

Artificial aging was carried out by thermocycling (Erios, São Paulo, São Paulo, Brazil) with 10,000 cycles at 5°C/55°C, with a dwell time of 25 sec and an exchange interval of 5 seconds, totaling 30 seconds [24].

### 2.2. Surface Treatments

After aging, the sixty-six specimens from each group (OBFa and FTKa) were subdivided into 3 groups (*n* = 22) according with the surface treatment executed (NT: no treatment/control; Abr: bur abrasion; or sand: sandblasting). The treatments were carried out on the smaller diameter base (2 mm) from each specimen. For the control group (NT) no treatment was performed, while for the group submitted to the abrasion treatment (Abr), the surface was colored with a 2B pencil (Faber-Castell, São Carlos, São Paulo, Brazil) to ensure homogeneous treatment over the entire surface. The abrasion was performed with a cylindrical bur (FG 2223, KG Sorensen, Cotia, São Paulo, Brazil), in a single direction movement across the surface until the graphite was fully removed. The treatment was carried out by a single operator, and the tips were replaced every 11 specimens. Finally, for the groups submitted to sandblasting (Sand), aluminum oxide was applied over the surface, also previously marked with the pencil. Particles with 50 *μ*m (BioArt, São Carlos, São Paulo, Brazil) were used with a pressure of 30 Psi, for 10 seconds, and distanced 1 cm from the specimen surface. Special care was taken to sweep the entire surface until removal of the graphite markings.

After surface treatments, all specimens were copiously washed and cleaned with 35% phosphoric acid (Ultra etch, Ultradent, Vivadent, Schann, Liechtenstein) for 60 seconds, followed by abundant washing and drying with air blast. Then, the universal adhesive system (Scotchbond Universal-3M ESPE, St. Paul, USA) was actively applied with a disposable tip for 20 seconds, followed by a 5 second air blast with a standardized distance of 15 mm for solvent evaporation, and cured for 10 sec with LED light (1400 mW/cm^2^, VALO, Ultradent Products, Inc. South Jordan, UT, USA).

**Figure 2:**
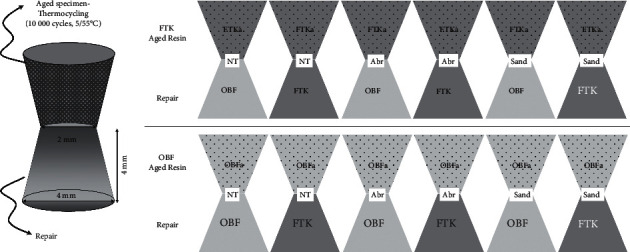
Schematic drawing of the specimens and group division.

In each subgroup, the specimens were again subdivided into 2 groups according to the material used for repair (conventional composite—FTK; bulk fill composite—OBF).  Figure 2 shows the chart of group division. For this, another truncated cone was built over the treated ones, with help of a second Teflon device as previously described by Pucci et al. [22], Feitosa et al. [25], and Zanatta et al. [23]. The repair was made either with conventional composite (FTK) or the bulk fill (OBF) one (Figure 2). The final specimen presented two truncated cones, adhered by the smaller diameter base, as shown in the schema of Figure 1.

### 2.3. Bond Strength Evaluation

The specimens were stored in relative humidity, at 37°C, for 24 hours, and then submitted to the microtensile strength test in a universal testing machine (EMIC DL 2000, São José dos Pinhais, Paraná, Brazil). A metallic device was used to adapt and align the specimens and perform the tensile strength as shown by Feitosa et al. [25]. The parameter used was a 10 kg load cell at a crosshead speed of 1 mm/min, according to the ISO 11405 standard, until failure of the set.

The bond strength was recorded in MPa by dividing the force (N) in the moment of the failure and the area of the adhesive interface (mm^2^). To calculate the bonding area, the diameter of the bonding interface was assessed with a digital caliper and used the circle area equation (*A*=*π* · *r*^2^).

Failure pattern was also analyzed at 20× magnification, under a stereomicroscope (Stereo Discovery V20, Zeiss, Göttingen, Germany), and the failures were classified as adhesive, when it occurred at the adhesive interface, cohesive, when occurred in composite resin (aged cone or repaired cone) or mixed.

### 2.4. Statistical Analysis

Data were checked for normality assumption (Kolmogorov–Smirnov test), and analysis of variance in two levels (2-way ANOVA) was performed for each aged composite resin (OBFa and FTKa), separately, considering surface treatment and composite type used for repair (OBF or FTK) as variables. Then, post hoc Tukey's test was performed with significance *p* < 0.05. Statistica for Windows (version 7, Statsoft Inc, Tulsa, USA) was used in the analysis.

## 3. Results

For the aged bulk fill composite (OBFa), the results of the two-way ANOVA test showed significant differences for the composite factor (*p*=0.0005) and the surface treatment (*p*=0.0003), but not for the interaction between them (*p*=0.3336).  Table 2 shows the mean values and the results of the statistical analysis considering the groups of aged bulk fill composite (OBFa). The Tukey test indicated that the aged bulk fill composite showed higher values of bond strength when repaired with the same material (OBFa/OBF > OBFa/FTK) and sandblasting and bur abrasion presented higher values compared to the control group, without surface treatment (Table 2).

For the aged conventional composite (FTKa), the results of the two-way ANOVA test showed no significant differences for the composite factor (*p*=0.6898) and the surface treatment (*p*=0.2368); however, there was differences between their interaction (*p*=0.0377). Only sandblasting presented lower values compared to bur abrasion for the group repaired with the bulk fill (FTKa/OBF). All other groups presented similar values.  Table 3 shows the mean values and the results of the statistical analysis considering the groups of aged conventional resin composite (FTKa).

**Figure 3:**
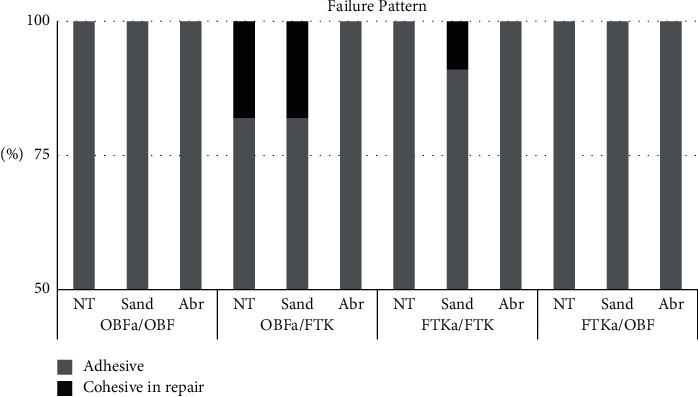
Frequency of the failure pattern.

Regarding failures, there was a predominance of adhesive one in all groups (Figure 3). Cohesive failures in the repair occurred only in the groups OBFa/FTK without surface treatment and sandblasted (18% each), and in the FTKa/FTK with sandblasting (9%).

## 4. Discussion

The results of this study indicated differences between the resin composites used for repair and surface treatments, particularly for the bulk fill one tested, therefore denying both null hypotheses. Repairs of partly defective restorations are indicated for resin-based composites (RBCs) aiming to increase the longevity of the restoration and consequently the tooth, being part of the minimally invasive dentistry philosophy [8]. The repairs involve partial removal of the defective part of the restoration, which is then replaced using a new material, and therefore, the knowledge of the composition of the resin composite is crucial for its success [26, 27]. One recent clinical trial showed that the performance of repaired restorations was similar to replaced ones in terms of marginal adaptation, secondary caries, color, and anatomy [28], encouraging its indication, even though literature is still controversial in the most effective technique to perform them, mainly regarding the requirement for surface treatments [8, 18, 29–35].

In repair procedures, the union between a new resin and the aged one occurs through the chemical union between the organic matrix and/or through mechanical retention [21]. The challenges associated to this procedure remains over the fact that the surface of the aged composites lacks any unreacted double bonds available for bonding to the new composite [18, 27]. The use of physical or chemical procedures, such as sandblasting, bur abrasion, acid etching (hydrofluoric or phosphoric acids) among others, aims to remove the outermost surface layer, which is altered by the saliva, oral films, and by the natural aging of the restoration, thus increasing the external layer surface energy and surface area and creating micro-irregularities [20].

Our results indicated that surface treatment was material dependent, as for the aged bulk fill composite tested, the sandblasting and bur abrasion promoted higher bond strength values compared to the single use of the adhesive system (Table 2), but this was not observed for the aged conventional composite (FTKa groups; Table 3). As the particle fillers type and volume were similar to both materials (Table 1), water sorption and degradation promoted by the aging protocol was not influenced by the inorganic phase, but the organic matrix [36]. The bulk fill composite tested presents different monomers such as AFM and AUDMA (Table 1) and could be exposed after bur abrasion or sandblasting favoring the formation of new chemical C-C bonds between the monomers from the aged and repair composite. These monomers are mainly responsible for volumetric and polymerization shrinkage reduction but also might have favored the formation of new cross-links between the adhesive and the repair material, especially AFM. According to the manufacture, the AFM monomer fragment during conversion and can then repolymerize in a lower stress state. Therefore, we can speculate that these fragments could be responsible to improve the bonding with the repair for the OBFa (aged) groups and also its superior effect when repaired with the same material (Table 2). Indeed, some previous works shows that better results are found when the material is repaired with the same one used in the first restoration [18, 30], but the challenge here remains, however, in the impossibility to identify which material was used in the restoration, if the repair was not made by the same operator.

Also, it should be pointed out that, for the conventional methacrylate composite (FTKa), no differences were found between groups (Table 3), indicating that although both resins have different monomeric composition (Table 1), there is compatibility between them, corroborating with the findings by Koç-Vural et al. [37], Bijelic-Donova et al. [38], and Medeiros et al. [39].

Still regarding surface treatment, the use of strong acids, such as hydrofluoric acid, has been reported as viable option; however, it may present a risk to the patient, due to contact with soft oral tissues and accidental swallowing in case when absolute isolation is not applied [40]. Phosphoric acid (35–37%) is safe for use inside the oral cavity, does not cause any superficial alteration in the resin, and is effective only as a superficial cleaning agent promoting the removal of organic contamination and smear layer [26, 39]. Regarding sandblasting with aluminum oxide, our data showed no difference with bur abrasion for all tested groups Thus, in terms of costs and technical approach, abrasion with a diamond tip is a more interesting option since it is a common tool for dentists and does not require investments in equipment to blast the aluminum oxide particles. Still, the literature reports that the safety of the blasting is questioned since the particles are small and can contaminate the aerosol which can be aspirated by both the dental team and the patient [33].

Regarding the adhesive system used in the present study, a universal adhesive (Scothbond Universal) was selected to simplify the steps. This adhesive is part of a new class of adhesive systems, called universal or multimodal, whose indication is for adhesion to tooth structures, ceramics, and other materials of indirect and also repairs. This system presents an organosilane in its composition potentially eliminating the silanization step when bonding to glass ceramics or resin composites [18, 41]. Since clinical procedures need to be as simple as possible to avoid mistakes during technical execution, the search for surface treatments to repairs that are simplified and safe is desirable, also justifying the use of a multimode adhesive.

Finally, the tensile stress test adopted in this study used the same parameter adopted by Pucci et al., Feitosa et al., and Zanatta et al. [22, 23, 25] and presented as an interesting option for tensile test with high predominance of adhesive failures, as shown by Figure 3, and without specimen loss or problems associated with conventional microtensile test. As an in vitro study, one of the main limitations refers to the aging protocol. In the oral cavity, restorative materials are subject to frequent pressure changes through chewing and biting, temperature changes, and electrolyte degradation due to the movement of salivary and microbiota fluids [24]. Commonly used in dentistry, the artificial aging of the samples in this study was done by thermocycling, following a previous protocol suggestive of 1 year of function [24, 42]. Future studies might include prolonged periods and evaluate the longevity of the repaired restoration. Also, the evaluation of different bulk fill composites with distinct composition, mechanical cycling, and clinical trials needs to be carried out in the future to validate the results found, as well as future analysis with analytic optimization techniques, such as described by Yadav [43].

## 5. Conclusions

The bulk fill resin composite tested present better repair performance when the same composite was used and sandblasting, or bur abrasion was performed. The conventional resin composite repair was less influenced by the material type and the surface treatment performed.

## Figures and Tables

**Table 1:** Composition of materials used in the study.

Material	Composition

**FTK**-filtek Z250 (3M ESPE)	Inorganic filler: zirconia and silica, (60% volume) with particle sizes ranging from 0,01 to 3,5 *μ*m
Shade-A2	
*Batch: 1824200198*	Contains bis-GMA, UDMA, and bis-EMA

**OBF**-Filtek one (3M ESPE)	Inorganic filler: combination of nonagglomerated/nonaggregated 20 nm silica particles, 4 to 11 nm nonagglomerated/nonaggregated zirconia particles, zirconia/silica nanoagglomerates and particles of particulate ytterbium trifluoride agglomerates of 100 nm, inorganic content of 58.5% (volume)
Shade-A2	
*Batch: N980337*	Contains AFM, AUDMA, UDMA, and DDDMA

Scothbond universal (3M ESPE)	Phosphate monomer (MDP), dimethacrylate resins, filler, HEMA, vitrebond^TM^ copolymer, alcohol, water, initiators, silane
*Batch: 1816000558*	

Phosphoric acid 35%,	Phosphoric acid 35%, thickener, dye and deionized water
Ultraetch (ultradent)	
*Batch: BFCCG*	

Bis-GMA: bisphenol-A glycidyl methacrylate; UDMA: dimethacrylate urethane; bis-EMA: bisphenol hydroxyethyl methacrylate; AFM: additional fragmentation monomer, AUDMA: aromatic dimethacrylate urethane; DDDMA: 1,12 dodecanediol dimethacrylate; HEMA: 2-hydroxyethyl methacrylate.

**Table 2:** Results of mean and standard deviation for the values of adhesive tensile strength and result of the Tukey's test for interaction between factors for the aged bulk fill groups (OBFa).

	Aged resin composite (OBFa)				
	Repair with OBF		Repair with FTK		Surface treatment factor
	Mean	DP	Mean	DP	

No treatment	9.23	(4.18) Aa	7.44	(3.64) Aa	8.33 (3.91) a
Sandblasting	14.64	(3.03) Ab	9.38	(3.63) Ba	12.01 (4.23) b
Abrasion	14.98	(2.69) Ab	11.53	(3.45) Aa	13.25 (3.49) b
Composite factor	12.95 (4.20) B		9.45 (3.84) A		

^*∗*^Uppercase letters show differences in the line for each resin composite. Lowercase letters show differences in the column for each surface treatment. Significant statistical difference between the groups (two-way ANOVA test followed by the Tukey's test, *p* < 0.05).

**Table 3:** Results of mean and standard deviation for the values of adhesive tensile strength and result of the Tukey test for interaction between factors for the aged conventional composite groups (FTKa).

	Aged conventional resin composite-FTKa				
	Repair with OBF		Repair with FTK		Surface treatment factor
	Mean	DP	Mean	DP	

No treatment	9.96	(2.27) Aab	9.52	(4.67) Aa	9.74 (3.57) a
Sandblasting	6.98	(3.13) Aa	11.09	(3.63) Aa	9.03 (3.91) a
Abrasion	12.38	(2.95) Ab	9.96	(5.25) Aa	11.17 (4.32) a
Composite factor	10.19 (4.44) A		9.77 (3.51) A		

^*∗*^Uppercase letters show differences in the line for each resin. Lowercase letters show differences in the column for each surface treatment. Significant statistical difference between the groups (two-way ANOVA test followed by the Tukey test, *p* < 0.05).

## Data Availability

The data used to support the findings of this study have been deposited in the local library repository and can be found at https://repositorio.unitau.br/jspui/handle/20.500.11874/4035. Also, data can be made available from the corresponding author upon request.
